# Distinguishing anchoring from confirmation bias in diagnostic vignette studies: methodological implications for clinical decision-making

**DOI:** 10.3389/fpubh.2026.1770198

**Published:** 2026-04-22

**Authors:** José A. Martínez, Laura Martínez

**Affiliations:** Technical University of Cartagena-Member of European University of Technology, Cartagena, Spain

**Keywords:** anchoring bias, cognitive bias, confirmation bias, decision-making, patient safety

## 1 Introduction

Cognitive biases in clinical decision-making have long been recognized as a major contributor to diagnostic error and patient harm, positioning them as a central concern in public health and healthcare quality research. Diagnostic error represents a substantial source of preventable patient harm, and cognitive biases have been repeatedly implicated as key contributors to these failures in clinical reasoning, particularly in internal medicine (1–5). Experimental vignette studies have emerged as a cornerstone methodology for examining how specific informational cues influence physicians' diagnostic judgments under controlled conditions.

Diagnostic vignettes are structured clinical scenarios, typically text-based descriptions of patients presenting with symptoms, history, and examination findings, designed to simulate real-world clinical encounters while allowing systematic manipulation of key variables (6, 7). These methodologies offer a hybrid approach that combines the internal validity of experimental designs with aspects of the external validity associated with survey research, enabling researchers to isolate and test specific hypotheses about clinical judgment (6–8). Within this literature, patient-provided diagnostic suggestions have received growing attention, given their increasing prevalence in real-world clinical encounters facilitated by online health information and their potential to shape clinicians' reasoning processes (1, 3).

In this context, Sánchez Ordóñez and colleagues present compelling experimental evidence showing that patient-suggested diagnoses can significantly influence physicians' diagnostic decisions (β = 1.396, *p* = 0.016), highlighting a mechanism with clear implications for patient safety and clinical training (1). Their findings contribute to a robust body of research demonstrating that diagnostic reasoning is not purely analytical but is susceptible to contextual and cognitive influences. However, while the empirical effect they document is convincing, we contend that the interpretation of the underlying cognitive mechanism warrants closer methodological scrutiny. We emphasize at the outset that our critique concerns the interpretation of the cognitive mechanism rather than the validity of the empirical findings themselves.

Reviews of cognitive diagnostic error underscore that premature closure and related biases are more frequent drivers of diagnostic failure than mere knowledge deficits, highlighting the need to understand the mechanisms underlying biased reasoning (3, 4). Specifically, the experimental design employed may conflate two conceptually distinct cognitive biases: anchoring and confirmation bias. Although both biases can lead to similar observable outcomes -persistence of an initial diagnostic impression despite contradictory evidence-, they arise from different psychological processes and, crucially, respond to different debiasing strategies (9–11). From a public health perspective, failing to distinguish between these mechanisms risks misinforming intervention design and scaling ineffective solutions in clinical practice.

The purpose of this opinion paper is therefore not to dispute the empirical findings of Sánchez Ordóñez et al., but to argue that greater conceptual and methodological precision is required to disentangle anchoring from confirmation bias in diagnostic vignette studies. Clarifying this distinction is essential for advancing theory, improving experimental validity, and designing targeted interventions to reduce diagnostic error at scale.

## 2 Conceptual framework: vignette methodologies and cognitive bias mechanisms

### 2.1 Diagnostic vignette studies: definition and methodological functions

Before examining the specific distinction between anchoring and confirmation bias, it is essential to establish a working definition of diagnostic vignette methodologies and clarify their structural characteristics. Diagnostic vignettes are controlled clinical scenarios that present patient information in structured formats to evaluate clinical judgment and decision-making processes (6, 7). These scenarios typically include: (a) patient demographics and presenting complaints, (b) relevant medical history and risk factors, (c) physical examination findings, (d) diagnostic test results (when applicable), and (e) response options or opportunities for participants to formulate diagnostic hypotheses.

Vignette studies serve multiple methodological functions in clinical decision-making research. They enable systematic manipulation of specific clinical cues while holding other variables constant, facilitating causal inference about how particular information influences diagnostic judgments (6, 8). They overcome ethical constraints that would prevent experimental manipulation in real patient encounters, while maintaining sufficient realism to elicit clinically meaningful responses. As Evans et al. (6) note, well-designed vignettes can achieve high generalizability to real-world behavior when they test specific, theoretically grounded questions about clinical judgment.

The temporal structure of vignette presentation is particularly relevant to the current discussion. Vignettes may be presented: (a) as complete cases with all information available simultaneously, (b) sequentially, with information revealed in stages to simulate real-time clinical encounters, or (c) interactively, allowing participants to request specific tests or information (6, 7). This structural variation has direct implications for whether and how anchoring vs. confirmation bias can be distinguished, as the temporal ordering of cues and the opportunity for hypothesis-driven information gathering represent distinct cognitive processes (12, 13).

### 2.2 Anchoring bias in clinical contexts

Anchoring bias, as originally described by Tversky and Kahneman, refers to the disproportionate influence of an initial value or piece of information on subsequent judgments, even when that initial cue is arbitrary or normatively irrelevant (14). One of the defining characteristics of anchoring is that it does not require semantic or causal relevance: random numbers, irrelevant comparisons, or meaningless reference points can systematically shift estimates. This phenomenon operates through an adjustment-and-insufficiency mechanism; individuals make insufficient adjustments away from an initial anchor value when forming judgments (14).

In clinical contexts, anchoring has been documented when early information, such as a triage label, preliminary test result, or initial diagnostic impression from another clinician, exerts undue influence on later diagnostic reasoning (9, 10, 15). Crucially, anchoring operates primarily at the initial encoding and weighting stage of information processing. The anchor establishes a reference point that subsequent information is interpreted relative to, rather than fundamentally altering the search for or evaluation of evidence.

It is important to note, however, that anchoring does not operate exclusively as a source of bias. In diagnostic contexts, the deliberate provision of empirically grounded base rates as explicit reference points can serve a constructive function, guiding clinicians toward more calibrated probability estimates. Anchoring can constructively improve clinical judgment; when a base rate is set as a judgmental anchor, it counteracts base rate neglect by forcing the incorporation of information that is typically ignored. This approach leads to significantly more calibrated and accurate estimates (16). This dual role of anchoring, as both a potential source of error and a potential debiasing instrument, has direct implications for the design of experimental vignettes and clinical decision support systems, and should be considered when interpreting the effects of anchoring manipulations in empirical studies.

### 2.3 Confirmation bias and hypothesis-driven processing

Confirmation bias, by contrast, involves a qualitatively different cognitive architecture. Rather than overweighting an initial cue *per se*, confirmation bias reflects the selective search for, interpretation of, and weighting of evidence that supports an activated hypothesis, coupled with the neglect or discounting of disconfirming information (17). In diagnostic reasoning, confirmation bias manifests when clinicians prematurely commit to a diagnostic hypothesis and subsequently preferentially seek tests, symptoms, or interpretations that reinforce that hypothesis.

The psychological mechanisms underlying confirmation bias are distinct from anchoring. Confirmation bias operates through hypothesis-driven attention allocation and asymmetric evidence evaluation (11, 17). Once a hypothesis is activated, it guides information search strategies; clinicians ask questions and order tests that would confirm the hypothesis while neglecting alternatives (12, 13). Moreover, ambiguous evidence is interpreted in ways that support the activated hypothesis, and disconfirming evidence is subjected to greater scrutiny or discounted entirely (17).

The distinction between these mechanisms aligns with broader frameworks of diagnostic reasoning. The hypothetico-deductive model posits that clinicians generate diagnostic hypotheses early in clinical encounters and then engage in hypothesis testing through iterative cycles of cue interpretation and hypothesis evaluation (12, 13, 18). Confirmation bias represents a failure mode within this process: hypothesis generation occurs, but hypothesis testing becomes asymmetric. Anchoring, conversely, may occur before hypothesis generation, biasing the initial interpretation of presenting information.

While anchoring and confirmation bias often co-occur in real-world clinical settings, their conceptual distinction is well established in cognitive psychology and has been empirically demonstrated in controlled studies (9, 11, 19). Anchoring primarily concerns the initial weighting of information, whereas confirmation bias concerns subsequent information processing. This distinction has direct implications for experimental design and intervention development.

### 2.4 Situating biases within dual-process theory

To further clarify the theoretical framework, it is instructive to situate anchoring and confirmation bias within dual-process models of cognition. Dual-process theory distinguishes between System 1 (intuitive, rapid, pattern-based) and System 2 (analytical, deliberative, rule-based) reasoning (20). Both anchoring and confirmation bias can manifest in either system, but their operation differs substantively.

Anchoring often operates through System 1 processes; the initial cue automatically activates related concepts and establishes a reference frame before deliberate reasoning begins (14). However, insufficient adjustment away from the anchor persists even when System 2 is engaged, suggesting that anchoring affects both intuitive and analytical processing.

Confirmation bias similarly spans both systems. Pattern recognition (System 1) may lead to premature diagnostic closure, while analytical reasoning (System 2) can be corrupted by hypothesis-driven selective information gathering (11, 13). The persistence of confirmation bias even among experienced clinicians engaging in deliberate reasoning underscores that it is not simply a failure to activate System 2, but rather a systematic distortion of how hypotheses guide evidence evaluation (19, 21).

## 3 Application to the current study and broader empirical literature

In the study under discussion, the patient's suggestion of a serious diagnosis constitutes a highly salient and clinically meaningful cue. Unlike a neutral anchor (such as an arbitrary numerical value or semantically unrelated reference point), such a suggestion directly activates a specific disease hypothesis. Consequently, any observed persistence of that hypothesis in the face of contradictory evidence could plausibly reflect confirmation bias rather than -or in addition to- anchoring.

This interpretation is consistent with experimental work demonstrating that salient distracting clinical features can strongly attract physicians' attention toward particular disease hypotheses and thereby misdirect diagnostic reasoning, especially in complex cases (22). In vignette-based research, such salient cues behave less like arbitrary anchors and more like meaningful hypothesis triggers, activating confirmation-driven information processing (21, 22). Mamede and colleagues have shown that when clinicians encounter salient features characteristic of a particular disease, they are more likely to prematurely commit to that diagnosis and engage in confirmatory rather than discriminatory hypothesis testing (22).

The authors themselves note that clinicians may maintain initial impressions despite conflicting data, a pattern consistent with both mechanisms but insufficient to distinguish between them empirically. Zwaan et al.'s (21) vignette study examining bias recognition among clinicians found that even experts in diagnostic error frequently disagree on which specific biases are present in a given case, underscoring the need for more precise operationalizations of constructs such as anchoring and confirmation bias.

A critical methodological limitation in the current design is the absence of a baseline assessment of clinicians' diagnostic hypotheses prior to the experimental manipulation. Without establishing what physicians suspected before encountering the patient's suggestion, it is impossible to determine whether the suggestion functioned as an anchor that shifted initial judgments or as a hypothesis-priming cue that triggered confirmatory information processing. Did the patient suggestion change physicians' initial impressions (anchoring), or did it activate a hypothesis that then guided selective evidence evaluation (confirmation)? The current design cannot definitively adjudicate between these alternatives.

This ambiguity extends across much of the vignette-based cognitive bias literature. A recent systematic review of educational interventions targeting cognitive bias in diagnosis found that many studies invoke multiple bias labels without clear operational definitions or mechanisms for distinguishing between them (23).

## 4 Methodological recommendations and future directions

### 4.1 Factorial experimental designs for bias disambiguation

Future vignette studies should adopt factorial experimental designs capable of orthogonally manipulating anchor neutrality and hypothesis activation. For example, a 2 × 2 design could independently vary: (Factor 1) whether an initial cue is semantically neutral (e.g., waiting time, administrative information) vs. hypothesis-relevant (e.g., patient-suggested diagnosis), and (Factor 2) whether participants are prompted to generate diagnostic hypotheses before vs. after receiving the cue.

Neutral anchors in clinical contexts could include arbitrary numerical values (e.g., “you are the 47th patient today”), unrelated temporal information (e.g., “appointment scheduled 3 weeks ago”), or administrative details (e.g., “referred from clinic A vs. clinic B”), cues semantically unrelated to any specific diagnosis that thus isolate pure anchoring effects.

Hypothesis activation can be manipulated through several approaches: (a) explicit diagnostic suggestions from patients (as in the focal study), (b) diagnostic labels from prior clinical encounters, (c) preliminary test results suggestive of specific diseases, or (d) salient distracting features characteristic of particular conditions. Each represents a clinically meaningful cue that activates a specific disease hypothesis.

This 2 × 2 design would yield four conditions: (i) neutral anchor with pre-exposure hypothesis generation, (ii) neutral anchor with post-exposure hypothesis generation, (iii) hypothesis-activating cue with pre-exposure hypothesis generation, and (iv) hypothesis-activating cue with post-exposure hypothesis generation. Comparing conditions (i) and (ii) would isolate pure anchoring effects (does an arbitrary cue shift judgments even when hypotheses are not yet activated?), while comparing conditions (iii) and (iv) would assess whether a clinically meaningful cue functions differently when it precedes vs. follows hypothesis generation.

Including a true control condition (no anchor, no hypothesis-activating cue) would allow researchers to calculate main effects and interactions, clarifying whether diagnostic distortions arise from anchoring, confirmation bias, or their synergistic combination. Prior vignette studies that manipulated salient distracting features and measured subsequent information-seeking behavior illustrate how factorial designs can reveal mechanism-specific effects beyond what is apparent from final diagnoses alone (22).

### 4.2 Process-tracing methods for direct evidence of confirmatory reasoning

Incorporating process-tracing methodologies would provide more direct evidence of the cognitive mechanisms underlying diagnostic errors. Process tracing involves systematic examination of intermediate steps in a decision process; the information sought, the sequence of hypothesis generation and revision, and the allocation of attention to confirming vs. disconfirming evidence (12, 13, 24). In diagnostic vignette studies, process tracing can be operationalized through several approaches.

Test-ordering patterns represent one concrete measure. If clinicians disproportionately request tests that would support a patient-suggested diagnosis while neglecting tests that would rule out alternatives, this asymmetry would directly implicate confirmation bias rather than simple anchoring (12, 22). Response time measures can also be informative: longer deliberation times coupled with unchanged diagnoses may indicate effortful attempts to justify an anchored impression, while rapid convergence on the anchored diagnosis suggests less analytical processing.

Information-seeking sequences in interactive vignettes provide particularly strong evidence. If participants are allowed to request additional patient history, physical examination findings, or test results, the pattern of requests can reveal whether they are engaging in hypothesis-testing (requesting discriminatory information) vs. hypothesis-confirmation (requesting only supportive information) (12, 13, 25).

Think-aloud protocols represent another process-tracing approach, though they require careful analysis to distinguish between different bias mechanisms. Verbal reports revealing selective attention to confirmatory evidence, dismissal of disconfirming findings, or failure to generate alternative hypotheses would strongly implicate confirmation bias. In contrast, verbal reports indicating that an initial cue “frames” subsequent information but without active hypothesis-driven selective processing would be more consistent with anchoring.

### 4.3 Translational considerations: ecological validity and real-world implementation

While the preceding discussion has focused primarily on construct validity and theoretical precision, it is essential to consider how vignette-based findings translate to real-world clinical environments. Ecological validity -the extent to which experimental findings generalize to authentic clinical settings- remains a central concern for vignette methodologies (6–8, 26).

Several features of real clinical environments may amplify or attenuate the cognitive biases observed in vignettes. Time pressure, cognitive load from managing multiple patients, interruptions, and fatigue are pervasive in clinical practice and have been shown to increase reliance on heuristic reasoning and vulnerability to bias (26, 27). Vignettes, typically completed without these stressors, may underestimate the magnitude of bias effects in authentic practice. Conversely, the opportunity for real-time patient interaction, physical examination, and consultation with colleagues may provide naturalistic ‘forcing functions' that interrupt biased reasoning, opportunities not available in static vignettes (2, 4).

Electronic health record (EHR) interfaces represent a particularly important ecological consideration. Modern EHRs prominently display preliminary diagnoses, triage classifications, referral reasons, and prior clinician assessments, all potential sources of anchoring or hypothesis activation (15). Whether these interface elements function as neutral anchors or as hypothesis-priming cues remains an open empirical question with direct implications for health information technology design. If EHR interfaces systematically activate confirmation bias, they may inadvertently amplify diagnostic error rather than reduce it.

Several strategies could enhance the ecological validity of vignette-based bias research while maintaining experimental control. Adaptive vignettes that dynamically modify scenarios based on participant choices can better simulate the contingent nature of real clinical reasoning (7, 10). Embedding vignettes within simulated EHR interfaces replicates the informational environment clinicians actually navigate. Team-based vignette studies, where multiple clinicians collaborate on case diagnosis, could assess whether cognitive biases persist, amplify, or attenuate in group decision-making contexts (26).

### 4.4 Implications for debiasing interventions and health systems

The distinction between anchoring and confirmation bias has direct implications for intervention design. These biases are associated with different cognitive architectures and respond to different debiasing strategies. Anchoring may be mitigated through relatively straightforward interventions, such as structured delays before final decisions, presentation of alternative reference points, or forced consideration of multiple initial hypotheses (2, 4). Cognitive forcing strategies that require clinicians to explicitly generate alternative diagnoses before committing to a primary impression have shown promise in reducing anchoring effects (4, 23, 28).

Confirmation bias, however, often requires more cognitively demanding strategies. Guided reflection -systematic consideration of alternative diagnoses with mentorship or structured prompts- represents one evidence-based approach (4, 23). The “consider-the-opposite” technique, where clinicians deliberately seek disconfirming evidence for their leading diagnosis, directly targets the asymmetric evidence evaluation characteristic of confirmation bias (4, 23). Differential diagnosis checklists and Bayesian reasoning frameworks may also reduce confirmatory processing by making alternative hypotheses salient and encouraging quantitative probability assessment (15).

A growing body of evidence on educational debiasing initiatives suggests that structured reflection and hypothesis-comparison strategies can improve diagnostic accuracy, but their effectiveness depends critically on alignment between the targeted mechanism and the intervention design (4, 23). Teaching clinicians to resist arbitrary anchors will yield suboptimal returns if the primary problem is hypothesis-driven confirmatory testing. Conversely, imposing cognitively demanding reflective strategies may be unnecessary, and potentially counterproductive given time constraints, if simple anchoring effects predominate.

Ludolph and Schulz examined debiasing strategies across 87 health-related judgment studies and found that the majority of interventions were at least partially effective, while identifying a lack of conceptual rigor in the underlying terminology as a key challenge (29). This observation directly reinforces the importance of distinguishing anchoring from confirmation bias not only for theoretical precision but also for practical intervention design. A complementary perspective is offered by research on individual differences in judgment quality. Work by Tetlock and colleagues, drawing on large-scale forecasting research, indicates that traits such as actively open-minded thinking and numerical reasoning skills are associated with more calibrated judgments and greater resistance to common heuristic errors (30). Such inter-individual variability may partly account for heterogeneous responses to standardized debiasing programs and highlights the potential value of assessing and targeting these modifiable cognitive dispositions in clinical education.

From a health systems perspective, recent frameworks for cognitive debiasing emphasize the need to combine individual-level educational strategies with system-level “forcing functions” that reshape decision environments (2, 4). Decision support systems that automatically generate differential diagnoses, EHR interfaces that de-emphasize preliminary impressions from earlier encounters, and team structures that build in diagnostic ‘checks' represent systemic approaches to bias reduction (2, 4, 15). However, these interventions must be grounded in accurate understanding of underlying mechanisms.

From a population health perspective, the stakes are high. Large-scale training programs, clinical guidelines, and digital interventions are increasingly deployed to reduce diagnostic error. If these interventions are based on an incorrect diagnosis of the underlying cognitive mechanism, their effectiveness will be limited or null. The substantial investment in diagnostic safety initiatives demands methodological rigor in identifying the specific cognitive processes that need to be targeted.

## 5 Conclusion

The work of Sánchez Ordóñez et al. (1) represents an important empirical contribution to understanding how patient-suggested diagnoses influence clinical decision-making. We have argued, however, that the field would benefit from greater methodological precision in distinguishing anchoring from confirmation bias, both in this study and in vignette-based cognitive bias research more broadly. This distinction is not merely conceptual; it has direct implications for how we design experiments, interpret findings, and develop interventions.

We have outlined concrete methodological approaches; factorial designs that orthogonally manipulate anchor type and hypothesis activation, process-tracing methods that reveal information-seeking patterns, and adaptive vignettes that enhance ecological validity, that can advance this research agenda. We have also situated these methodological recommendations within broader theoretical frameworks of diagnostic reasoning and dual-process cognition, demonstrating that anchoring and confirmation bias represent distinct failures in different cognitive systems.

The ecological validity considerations we have raised underscore that laboratory findings must ultimately translate to real clinical environments characterized by time pressure, cognitive load, team dynamics, and complex health information technology systems. The challenge for future research is to maintain experimental control while incorporating these contextual factors in ways that enhance rather than compromise the inferential value of vignette studies.

Achieving methodological precision in cognitive bias research is not an academic luxury but a prerequisite for designing effective, scalable interventions to improve diagnostic accuracy and patient safety. As vignette-based research continues to inform public health policy, clinical education, and health systems redesign, refining our conceptual frameworks and experimental methods will be essential to ensure that empirical insights translate into meaningful improvements in care. The diagnostic errors that harm patients demand nothing less than our most rigorous science.
